# Horizontal Ridge Expansion: A Comparative Study of Motorized Ridge Expanders Versus Osseodensification Techniques

**DOI:** 10.7759/cureus.73875

**Published:** 2024-11-17

**Authors:** Tushar, Ananya, Priya Rani, Jayant Prakash, Jayaprakash M B, Poonam Kumari Jayaprakash

**Affiliations:** 1 Prosthodontics, Rajendra Institute of Medical Sciences, Ranchi, IND; 2 Orthodontics, Rajendra Institute of Medical Sciences, Ranchi, IND

**Keywords:** atrophic bone, implant, isq value, motorized ridge expanders, osseodensification, primary stability

## Abstract

Background: Adequate horizontal and vertical bone volume is an ideal prerequisite to achieving good primary stability, osseointegration, and long-term survival of an implant. Several techniques are available to achieve adequate bone volume for implant placement. Among the various non-subtractive methods, ridge expansion with motorized bone expanders is a commonly used method to expand bone volume in the anterior maxilla. At the same time, osseodensification is another non-subtractive method that aims to improve primary stability.

Aim: This study aimed to compare the expansion achieved by the two most commonly used expansion methods, i.e., motorized bone expanders technique and a relatively newer technique of osseodensification suggested by Densah Bur (Jackson, Michigan, United States), for expanding the alveolar ridge in the anterior maxillary region.

Materials and methods: A total of 30 implants were placed in the anterior maxillary region in 15 patients. Fifteen implants were placed with the bone expander method (Dentium Ridge Spreader (RS) kit, Cypress, California, United States) and 15 with the osseodensification method (Densah® Bur). At least two implants were placed in each patient. The implants were placed in the first and second quadrants of the same patients in whom one of the methods was performed. Alveolar ridge expansion was determined using pre-op and post-op cone beam computed tomography (CBCT) instantly before implant placement and after six months. Primary stability was also analyzed using Osstell (Gothenburg, Sweden) immediately after implant placement and after one month and secondary stability after six months in implant stability quotient (ISQ). These parameters were used to assess which method promoted greater alveolar ridge expansion and primary stability.

Result: The test for normality was performed using the Shapiro-Wilk test between the pre- and post-treatment data between group A (motorized ridge expander) and group B (osseodensification technique). There were p-values of 0.737 and 0.429, which were not significant. Bone thickness was measured both preoperatively and postoperatively among the groups. The mean bone thickness in group A was 4.37 mm preoperatively and 5.8 mm postoperatively. The similarity between the preoperative and postoperative bone thickness was 4.336 mm and 5.24 mm, respectively. For the bone measurements, the p-value <0.001 is highly significant. On further analysis, the p-value for the paired samples t-test for group A postoperative bone thickness and group B postoperative bone thickness was 0.894, and the p-value for the t-test for group A postoperative bone thickness and group B postoperative bone thickness was 0.955. ISQ was also analyzed. In group A, the mean ISQ one month postoperatively was 53.2 and six months postoperatively was 73.4. In group B, the mean ISQ one month postoperatively was 65.4 and six months postoperatively was 76.2. The p-value for the ISQ of group A was 0.004, the ISQ of group B was 0.023, and the ISQ of groups A and B in the first month was 0.015. In the sixth month, the ISQ in group A and group B was 0.592.

Conclusion: According to the results of the study, motorized ridge expanders proved to be more effective in terms of ridge expansions and achieving better primary stability than the osseodensification technique.

## Introduction

The mechanical stability of the implants at the time of surgery is a crucial factor for the osseointegration of the implants. An insufficient amount of bone around the implants could negatively affect the implant stability. The most common technique to prepare the osteotomy for implant placement is surgical drilling of the bone, and ridge expansion with motorized drill expanders (Dentium Ridge Spreader (RS) kit, Cypress, California, United States) is one of the methods of ridge augmentation. Recently, a new drilling concept for implant placement has been introduced. Osseodensification, which is a non-extraction technique, increases the primary stability of implants by preservation and condensation through the compaction of autograft during osteotomy. The burs used for osseodensification may expand bone ridges less in width similar to the split-crest method, by compressing the bone along the inner surface of the implant osteotomy without cutting. However, the ridge expansion achieved with osseodensification is limited. Therefore, this study aimed to examine the expansion of the alveolar ridge and the stability of dental implants with the motorized ridge expanders compared to osseodensification drilling with multi-layer drills (Densah® Bur, Jackson, Michigan, United States) [1-3].

## Materials and methods

This study was conducted at the Institute of Technology and Sciences Dental College, Hospital and Research Center in Greater Noida, India. Approval to conduct the study was obtained from the Institutional Ethics Committee of the Institute of Technology and Sciences (approval number: ITSDCGN/2018/001).

In this study, 30 implants were placed in the atrophic anterior maxillae of 15 systematically healthy patients who required the replacement of missing teeth. Inclusion criteria included patients with missing maxillary anterior teeth in the first and second quadrants with insufficient horizontal width of >3-4 mm of bone. Exclusion criteria included patients with any medical history affecting bone healing and patients with smoking and chronic alcoholism. In each of the 15 patients, two implants were placed in the same arch but different quadrants with the two distinct techniques of bone expansion with group A being MRE (motorized ridge expander) and group B being OD (osseodensification).

Intraoral rinsing was done using a povidone-iodine germicide gargle of 2% (Betadine) before the surgical procedure. Local infiltration (2% lignocaine hydrochloride with 1 in 80000 adrenaline) was administered, and a mucoperiosteal incision was made on the crest of the alveolar ridge along with a relieving vertical incision on the buccal area followed by the elevation of full-thickness flap exposing the alveolar bone. Before implant placement, preoperative alveolar bone width was measured using pre-op cone beam computed tomography (CBCT) at a height of 2 mm from the crest. The implants were then placed on one of the sides, and ridge augmentation was done with the osseodensification method with the Densah® Bur kit as per the instructions of the manufacturer. On the opposite side, the implants were placed, and ridge augmentation was done with threaded motorized bone expanders (Dentium RS kit). The implants used for the study were ADIN Touareg S 3 mm and 3.5 mm implants (Afula, Israel).

Once the desired expansion was achieved in both techniques, implants of 3 and 3.5 mm were placed (ADIN implant system Touareg-STM, Afula, Israel). Post-expansion width was again determined after six months with a post-op CBCT. Expansions were only achieved till the buccal or lingual bone plate did not get fractured.

Implant stability quotient (ISQ) was assessed using Osstell ISQ (Osstell®, Gothenburg, Sweden) immediately after implant placement, after one month postoperatively, and then six months later.

## Results

The normality test was performed using the Shapiro-Wilk test between the pre- and post-treatment data between group A (osseodensification technique) and group B (motorized ridge expander). There were p-values of 0.737 and 0.429, which were not significant.

Bone thickness was measured preoperatively and postoperatively between the groups (Table 1). The mean bone thickness in group A was 4.37 mm preoperatively and 5.8 mm postoperatively. The similarity between the preoperative and postoperative bone thickness was 4.336 mm and 5.24 mm, respectively. For the bone measurements, the p-value <0.01 is highly significant.

**Table 1:** Mean bone thickness between and within groups MRE: motorized ridge expanders; OD: osseodensification; **: statistically significant

	Group A (MRE)	Group B (OD)	P-value
Preoperative	4.37	4.336	0.894
Postoperative	5.8	5.34	0.955
P-value	0.001**	0.008**	

On further analysis, the p-value for the paired samples t-test for group A by bone thickness and group B by bone thickness was 0.894, and the p-value for the paired samples t-test for group A by bone thickness and group B by bone thickness was 0.955.

**Figure 1:**
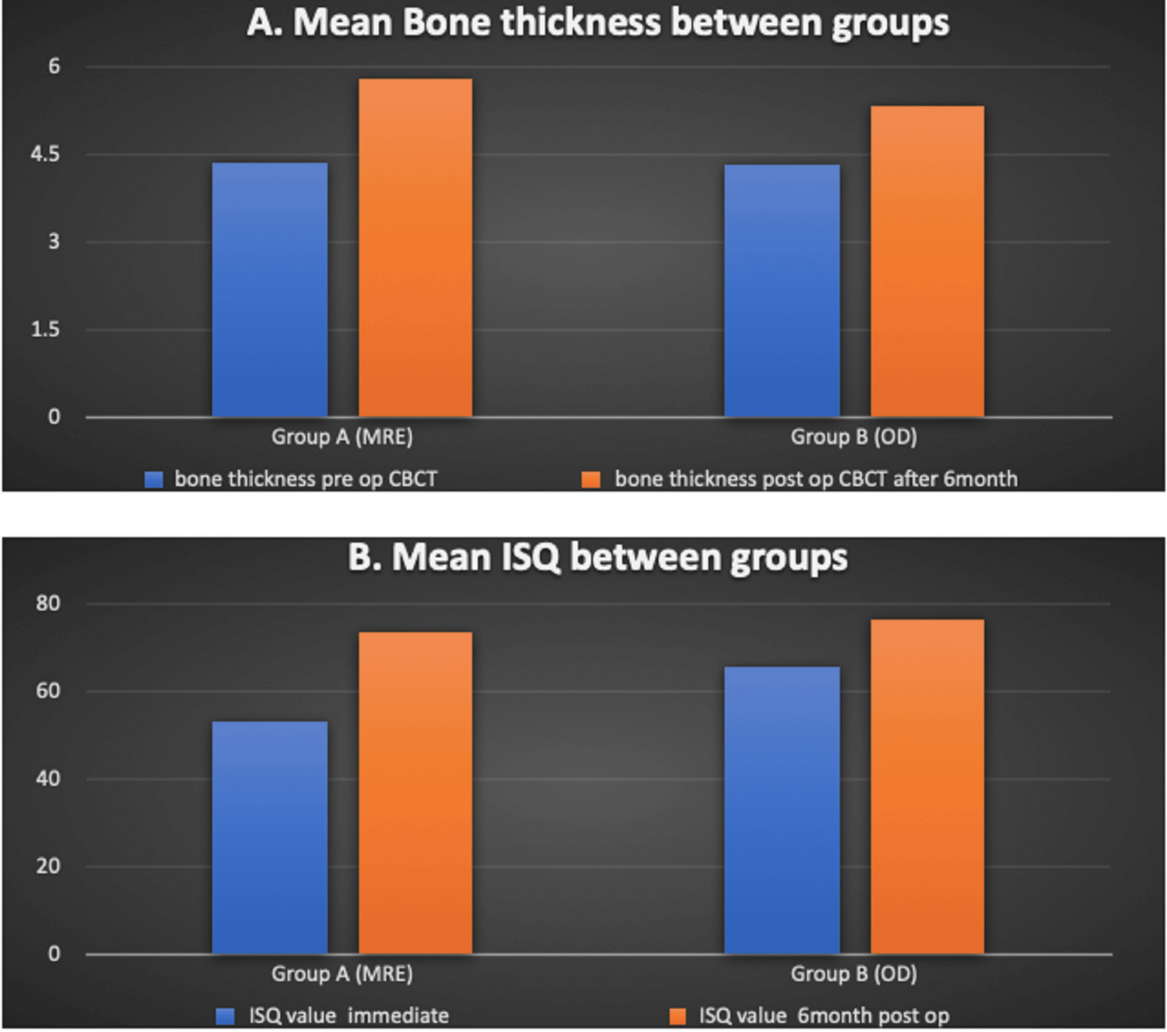
(A) Mean bone thickness and (B) ISQ between both groups MRE: motorized ridge expanders; OD: osseodensification; **: statistically significant

The ISQ was also analyzed. In group A, the mean ISQ one month postoperatively was 53.2 and six months postoperatively was 73.4. In group B, the mean ISQ one month postoperatively was 65.4 and six months postoperatively was 76.2 (Table 2, Figure 1).

**Table 2:** ISQ between and within groups MRE: motorized ridge expanders; OD: osseodensification; ISQ: implant stability quotient; **: statistically significant

	Group A (MRE)	Group B (OD)	P-value
One month post-op	53.2	65.4	0.015**
Six months post-op	73.4	76.2	0.592
P-value	0.004**	0.023**	

The p-value for group A ISQ was 0.004 and for group B ISQ was 0.023. Group A and B ISQ in the first month was 0.015. Sixth-month ISQ for groups A and B was 0.592.

**Figure 2:**
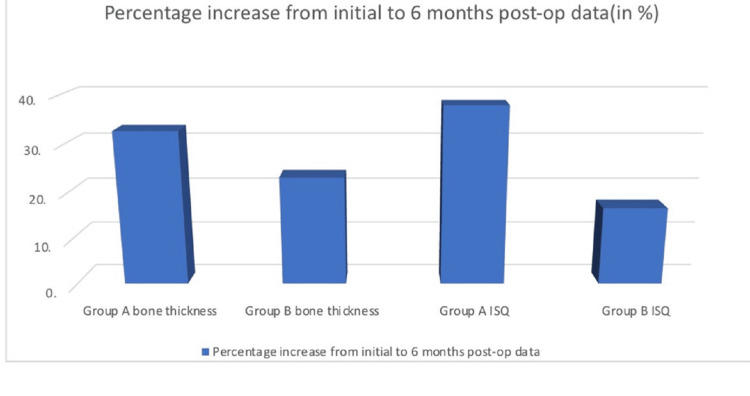
Percentage increase of bone thickness and ISQ after six months of follow-up ISQ: implant stability quotient; **: statistically significant

The percentage changes before and after surgery were evaluated. In group A, bone thickness increased by 32.72% compared to 23.04% in group B. Using the baseline ISQ data one month after implantation, increases of 37.97% in group A and 16.51% in group B were observed in the six months after surgery (Figure 2).

## Discussion

It is well known that when the alveolar ridge width is less than 5 mm, augmentation is required to obtain a dental implant with 1.5-2 mm of healthy bone around the implant [4]. Placing implants in these areas with insufficient ridge width can lead to the following problems: labial dehiscence, increased likelihood of peri-implantitis, unaesthetic exposure of the metal through the gingiva, and introduction of off-center loading [5].

These issues can be corrected by augmenting the bone either by grafting or by atraumatic ridge expansion techniques (ARETs). However, bone grafting requires an additional surgical site if it is autogenous bone grafting. Hence, horizontal bone augmentation along with implant placement in a single surgical procedure enhances the treatment outcome by expanding bone volume [6]. ARETs virtually reduce cost and time and eliminate the two surgical procedures [6].

Various ARETs are mentioned in the literature, of which the most common technique of ridge expansion is performed with osteotomes, but osteotomes cannot control the amount of expansion and the pressure required for expansion. The second most common technique is the use of a motorized bone expander, which can control the amount of pressure and achieve the desired expansion [7]. Of the various techniques, a new technique has been presented in the literature to increase bone density and achieve some ridge widening, namely, osseodensification [8-10]. Studies have shown that osseodensification gives better primary and secondary stability and greater width expansion in atrophic bone compared to the osteotome technique [10]. As the motorized expander is far superior to the osteotome technique, this study was designed to verify the stability of the implant and the extent of ridge expansion achieved with the osseodensification technique and the motorized ridge expansion techniques [7].

Our study provides different results than the existing literature [7-10]. According to our study, although there is no great change in the primary stability of the implant at the time of surgery, the results after six months show that motorized ridge expanders provide better stability than osseodensification and, in terms of the extent of expansion achieved, motorized ridge expanders provide more or less better results than osseodensification both at the time of surgery and six months after surgery.

## Conclusions

The current study supports the usage of motorized ridge expanders for horizontal ridge expansion in atrophic areas compared with osseodensification as expansion achieved with osseodensification is limited. There were certain limitations of the current study such as the small sample size, whether the full potential of ridge split surgery could be achieved or not, how much expansion could be achieved before the cortical plate fractured, etc. Hence, there is a need to conduct future studies in this field with a larger sample size covering the limitations.

## References

[1] Huwais S, Meyer EG (2017). A novel osseous densification approach in implant osteotomy preparation to increase biomechanical primary stability, bone mineral density, and bone-to-implant contact. Int J Oral Maxillofac Implants.

[2] Oliveira PG, Bergamo ET, Neiva R, Bonfante EA, Witek L, Tovar N, Coelho PG (2018). Osseodensification outperforms conventional implant subtractive instrumentation: a study in sheep. Mater Sci Eng C Mater Biol Appl.

[3] Kanathila H, Pangi A (20181). An insight into the concept of osseodensification-enhancing the implant stability and success. J Clin Diagn Res.

[4] Goyal S, Iyer S (2009). Bone manipulation techniques. Int J Clin Implant Dent.

[5] Khairnar MS, Khairnar D, Bakshi K (2014). Modified ridge splitting and bone expansion osteotomy for placement of dental implant in esthetic zone. Contemp Clin Dent.

[6] Tian JH, Neiva R, Coelho PG (2019). Alveolar ridge expansion: comparison of osseodensification and conventional osteotome techniques. J Craniofac Surg.

[7] Jha N, Choi EH, Kaushik NK, Ryu JJ (2017). Types of devices used in ridge split procedure for alveolar bone expansion: a systematic review. PLoS One.

[8] Padhye NM, Padhye AM, Bhatavadekar NB (2020). Osseodensification -- a systematic review and qualitative analysis of published literature. J Oral Biol Craniofac Res.

[9] Gaspar J, Esteves T, Gaspar R, Rua J, João Mendes J (2018). Osseodensification for implant site preparation in the maxilla‐ a prospective study of 97 implants. Clin Oral Implant Res.

[10] Marão HF, Pimentel AC, Brandão TL (2019). Alveolar ridge expansion - comparative study of summers and osseodensification techniques. Clinical Oral Implants Research.

